# Involvement of FANCD2 in Energy Metabolism via ATP5α

**DOI:** 10.1038/s41598-017-05150-1

**Published:** 2017-07-07

**Authors:** Panneerselvam Jayabal, Chi Ma, Manoj Nepal, Yihang Shen, Raymond Che, James Turkson, Peiwen Fei

**Affiliations:** 1 University of Hawaii Cancer Center, Honolulu, HI USA; 2 Graduate Program of Molecular Biosciences and Bioengineering, University of Hawaii, Honolulu, HI USA

## Abstract

Growing evidence supports a general hypothesis that aging and cancer are diseases related to energy metabolism. However, the involvement of Fanconi Anemia (FA) signaling, a unique genetic model system for studying human aging or cancer, in energy metabolism remains elusive. Here, we report that FA complementation group D2 protein (FANCD2) functionally impacts mitochondrial ATP production through its interaction with ATP5α, whereas this relationship was not observed in the mutant FANCD2 (K561R)-carrying cells. Moreover, while ATP5α is present within the mitochondria in wild-type cells, it is instead located mostly outside in cells that carry the non-monoubiquitinated FANCD2. In addition, mitochondrial ATP production is significantly reduced in these cells, compared to those cells carrying wtFANCD2. We identified one region (AA42-72) of ATP5α, contributing to the interaction between ATP5α and FANCD2, which was confirmed by protein docking analysis. Further, we demonstrated that mtATP5α (∆AA42-72) showed an aberrant localization, and resulted in a decreased ATP production, similar to what was observed in non-monoubiquitinated FANCD2-carrying cells. Collectively, our study demonstrates a novel role of FANCD2 in governing cellular ATP production, and advances our understanding of how defective FA signaling contributes to aging and cancer at the energy metabolism level.

## Introduction

At the beginning of the twentieth century, Otto Warburg demonstrated the unique metabolic capabilities that cancer cells possess, preferentially utilizing glycolysis for energetic and anabolic purposes, whilst producing a large amount of lactic acid^1^. He defined this “aerobic glycolysis” and hypothesized that disturbances in mitochondrial activity played a specific, pathogenic role in cancer development^2^. To date, the roles of the mitochondria have been increasingly drawing attention, with hallmarks of almost every disease linked to improper mitochondrial function including FA^3, 4^. Additionally, although genomic abnormalities in cancer can often arise as an outcome of mitochondrial dysfunction, when established, genomic instability further contributes to mitochondrial dysfunction^5^.

Fanconi Anemia (FA) is a rare human disease with an early onset of aging, accompanying many developmental abnormalities and an extremely high incidence of both hematological and non-hematological malignancies^6–11^. At the cellular level, FA is characterized by chromosomal abnormalities and hypersensitivity to DNA crosslinking agents^6, 12, 13^. The similar sensitivity to DNA crosslinking agents of FA cells from at least 21 complementation groups known so far [FANC-A, B, C, D1, D2, E, F, G, I, J, L, M, N, O, P, Q, R, S, T, U and V^7, 10, 11, 14–24^], and common clinical symptoms suggest that the all FA proteins function in a common signaling transduction pathway (the FA pathway). This pathway is activated during DNA replication or upon DNA damage, especially when triggered by DNA crosslinking agents such as mitomycin C (MMC), diepoxybutane (DEB), and Cisplatin^25, 26^. Moreover, FANCD2 monoubiquitination at K561 works in conjunction with other FA and non-FA proteins^16, 18, 27–31^ to repair DNA damage. As FANCD2 appears to be the focus of the FA pathway^14^, monoubiquitinated (at K561) FANCD2 can act as a functional representative for the functions of activated FA signaling. Numerous studies have been conducted to determine the roles of this signaling network in human cancer development. However, it is rarely studied as to the involvement of this signaling in the mitochondrial function contributes to the understanding of cancer or aging associated with FA.

In this study, we report that FANCD2 contributes to the maintenance of mitochondrial function by partnering with ATP5α, a subunit of ATP synthase, located at the inner membrane of mitochondria^32^. These results demonstrate a previously unknown role of FANCD2 in governing cellular ATP production, advancing our understanding of how defective FA signaling contributes to aging and cancer through the scope of energy metabolism.

## Results

### Wild type FANCD2, not K561R FANCD2, interacts with ATP5α

**Figure 1:**
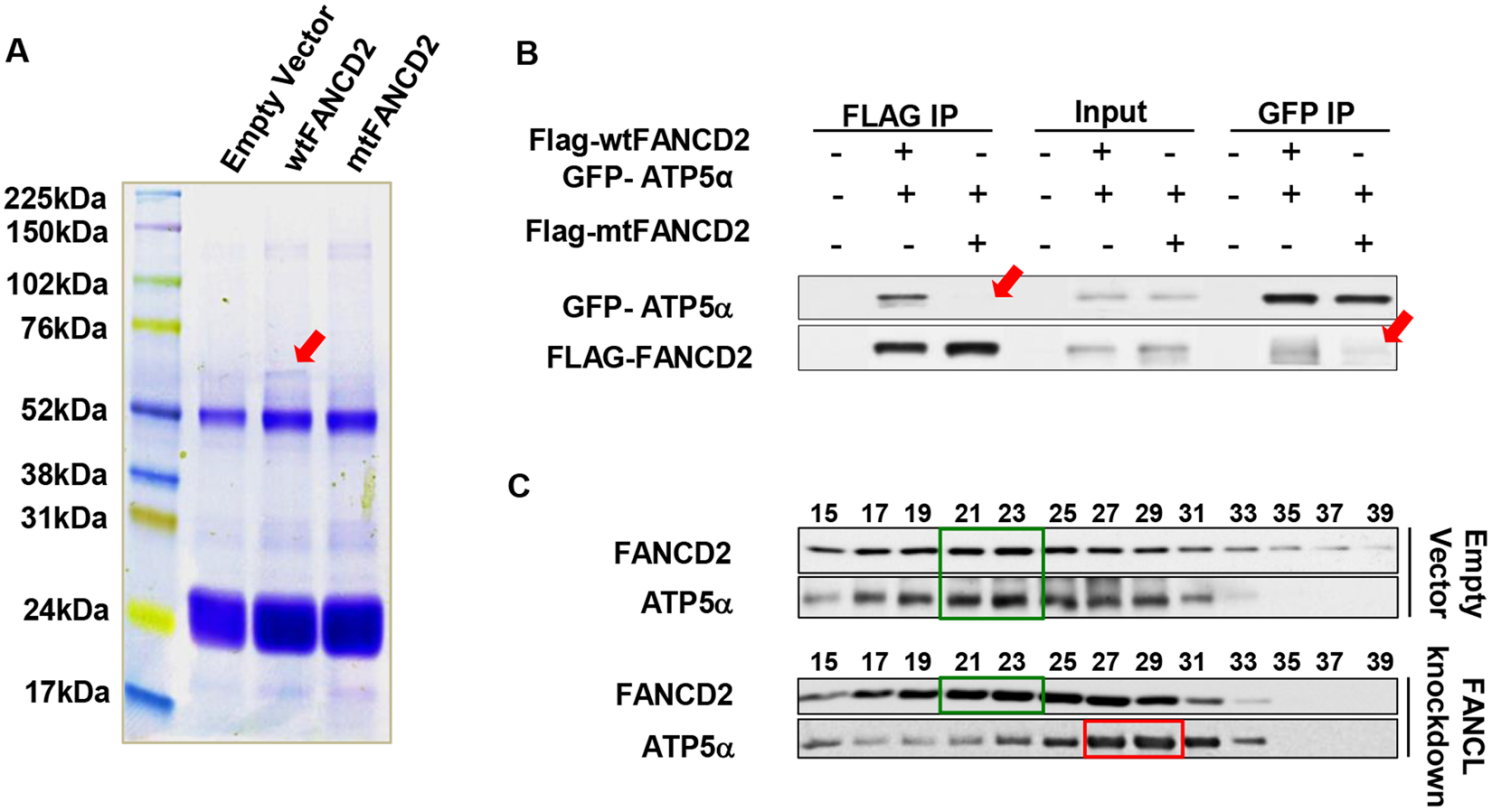
Interaction between FANCD2 and ATP5α. (**A**) Simple blue-staining of an 8–16% gradient gel shows “an additional extra band” in the “wtFANCD2”-lane but not in the other lanes. Flag-IP elutes were resolved in an 8–16% gradient gel, which were prepared from Flag-vector, Flag-wtFANCD2 or mtFANCD2-containing plasmids transfected 293T cells. “The extra band” pointed by a red arrowhead was sliced for mass spectrometry. (**B**) Confirmation of the interaction between monoubiquitinated FANCD2 and ATP5α. 293T cells were transiently co-transfected with GFP-fused ATP5α and Flag-wtFANCD2 or Flag-mtFANCD2 K561R. Cell lysate was prepared 48 hr post-transfection. Both Flag and GFP antibodies were used to perform reverse immunoprecipitation (IP) and Western blotting (WB). As red arrowheads indicated, Flag-mtFANCD2, unlike Flag-wtFANCD2, is not clearly associated with GFP-ATP5α (The equal transfection efficiency of 293T cells with GFP-fused ATP5α and Flag-wt or mt FANCD2 is shown in Supplementary Fig. 1A and B). (**C**) wtFANCD2 and ATP5α proteins peak at the same gel-filtration fractions prepared from control cells but not cells carrying silenced FANCL, which leads to a compromised FA-complex E3 and compromised monoubiquitination/activation of FANCD2. The sepharose 6B chromatography (gel filtration) was performed using U2OS cells stably transfected with empty vector or Lentivirus-FANCL-shRNA (Supplementary Fig. 1C). The peak of endogenous FANCD2 overlapped with the one of endogenous ATP5α protein in cells carrying a normal basal level of FANCD2 monoubiquitination, but not in the cells carrying a compromised basal level of FANCD2 monoubiquitination (resulting from silenced FANCL) (Fractions #15 and #39 correspond to the size markers of 2000 kDa and 669 kDa respectively).

By serendipity, we found that ATP5α was present in the protein pellets immunoprecipitated by FANCD2 antibodies from cells carrying a fully functioning FANCD2 via mass spectrometric analysis. However, this was not shown in the pellets generated from the cells possessing a mutant FANCD2 at K561 (Fig. 1A). This prompted us to study a potential interaction between FANCD2 and ATP5α, a subunit of the ATP synthase complex^32^. First, we conducted reverse immunoprecipitation and western blotting analyses to confirm their interaction. As shown in Fig. 1B (the transfection efficiency in Supplementary Fig. 1A and B), both Flag-wtFANCD2 and GFP-ATP5α can pull down each other in the lysates prepared from cells co-transfected with Flag-wtFANCD2 and GFP-ATP5α, but not the case for the cells carrying GFP-ATP5α and Flag-mtFANCD2 (K561R, leading to an inactivated form of FANCD2^33^). This observation was further supported by the studies that showed the same pattern of ATP5α distribution in gel filtration fractions prepared from the whole cell lysates of the control cells, compared to the corresponding cells harboring defective E3 FA complex (silenced FANCL) that is unable to monoubiquitinate FANCD2 (Fig. 1C and Supplementary Fig. 1C). Taken together, our results indicate that cells deficient in the activation of FANCD2 may have an improper function of ATP5α, thereby influencing mitochondrial function.

### Mitochondria in cells carrying a compromised level of monoubiquitinated FANCD2 are malfunctioned

**Figure 2:**
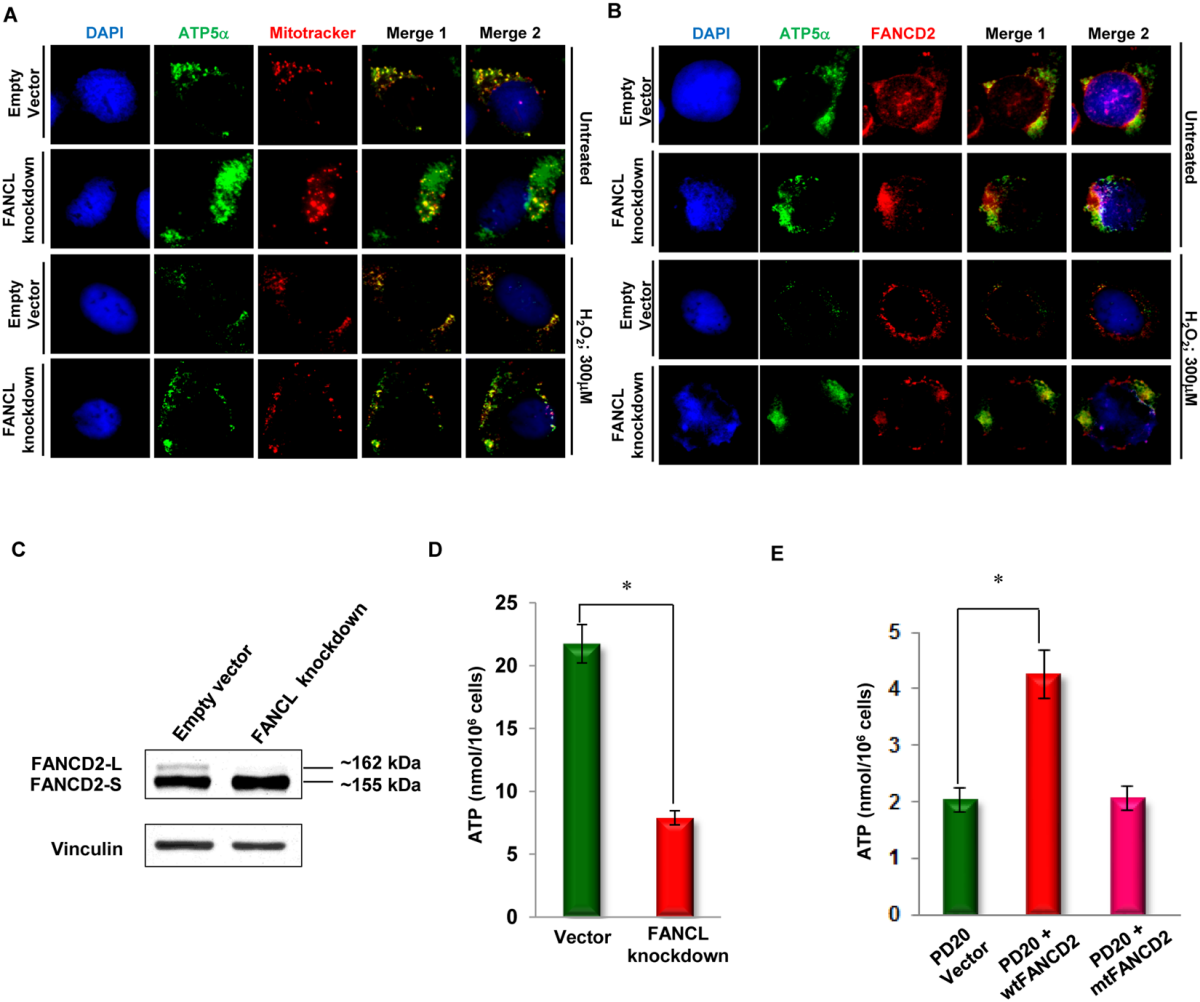
Cells carrying a compromised basal level of FANCD2 monoubiquitination harbor misplaced ATP5α and a reduced amount of ATP. (**A**) Co-localization of mitochondria with ATP5α protein in the control cells, but not in cells carrying silenced FANCL (a compromised basal level of FANCD2 monoubiquitination). U2OS stable cells carrying a normal basal level of FANCD2 monoubiquitination or a compromised basal level of FANCD2 monoubiquitination with or without H_2_O_2_ treatment were incubated with MitoTracker Red CMXRos (red fluorescence) and goat anti-ATP5α antibodies (green fluorescence) to identify the locations of the mitochondria and ATP5α respectively. Confocal microscopy analysis was performed using appropriate filters for visualization of red, green, or combined fluorescence. The images were taken at a magnification of 600X. (**B**) Co-localization of wtFANCD2 protein with ATP5α protein in the control cells, but not in cells carrying silenced FANCL (a compromised basal level of FANCD2 monoubiquitination). The same cells were incubated with anti-FANCD2 antibodies (red fluorescence) and goat anti-ATP5α antibodies (green fluorescence) to mark their cellular locations respectively in the presence or absence of H_2_O_2_ treatment. Confocal microscopy analysis was performed using appropriate filters for visualization of red, green, or combined fluorescence, and the images were taken at a magnification of 600X. (**C**) Conformation of the reduced basal level of monoubiquitnated FANCD2 in U2OS derivative cells harboring silenced FANCL (Supplementary Fig. 1C). FANCD2-L (Long form) is the monoubiquitinated form (about 162 kDa) and FANCD2-S (short form) is non-ubiquitinated form of FANCD2 protein (about 155 kDa)^52^. (**D**) ATP production in FANCD2 monoubiquitination-compromised cells was reduced compared to the control cells. Intracellular ATP production was measured by using the U2OS stable cell pair expressing FANCL at a normal or silenced level, in which the basal level of FANCD2 monoubiquitination was at the normal or low levels accordingly (Supplementary Fig. 1C and Fig. 2C). ATP production was significantly higher (*p* < 0.005) in the control cells compared to FANCL-silenced cells carrying a low basal-level of monoubiquitinated FANCD2. 1 × 10^6^ cells were lysed and intracellular ATP was determined with a colorimetric/fluorometric assay kit. (**E**) ATP production was low in PD20 cells (FANCD2−/−) stably expressing mtFANCD2 K561R or carrying the empty vector (control), compared to those cells carrying wtFANCD2. PD20 derivative cells were established, carrying wtFANCD2, mtFANCD2 or empty vector for control respectively (Supplementary Fig. 2). Intracellular ATP content was determined via the same kit used above (**C**). 1 × 10^6^ cells were lysed, and intracellular ATP content was determined. There is a significant reduction in the production of ATP in mtFANCD2-carrying cells or control cells compared to wtFANCD2-carrying PD20 cells (*p* < 0.005).

Monoubiquitinated FANCD2 has emerged to be a crucial center player in the FA signaling network. It appears to act as a multifunctional player, such as the recruitment of DNA polymerase eta for the earlier response to DNA damage compared to PCNA that we reported previously^34^. Therefore, we hypothesized that wtFANCD2 may help recruit ATP5α to function at mitochondria, in contrast to mutant FANCD2 (K561R-disrupting the monoubiquitination) that does not interact well with ATP5α (Fig. 1). To this end, we examined ATP5α’s compartmentation. We found that nearly a half of ATP5α proteins are localized outside mitochondria in both stressed (H_2_O_2_ treatment) and non-stressed cells carrying a compromised level of monoubiquitinated FANCD2 (via silenced FANCL shown in Supplementary Fig. 1C). This was compared to the similarly treated cells harboring a normal level of monoubiquitinated FANCD2 (control cells), in which all of the ATP5α proteins are localized in the mitochondria (Fig. 2A and C). This observation suggests that without monoubiquitinated FANCD2, the function of ATP synthase may be compromised, given the fact that ATP5α is one of its components^32^, and thus resulting in malfunctioned mitochondria. In addition, we detected the *in vivo* co-localization between FANCD2 and ATP5α to further support their interaction. We found that cells defective in the basal level of FANCD2 monoubiquitination, did not show a clear merge image as shown in control cells (Fig. 2B). Subsequently, we measured cellular ATP production in the same batch of cells carrying a normal or a compromised level of monoubiquitinated FANCD2 (Fig. 2C), because ATP production is directly related to the functional state of ATP synthase equipped at the inner membrane of mitochondria^35^. As shown in Fig. 2D, the amount of ATP is dramatically decreased in cells harboring inactivated FANCD2 comparing to those cells carrying a normal level of FANCD2 monoubiquitination. This was also shown in three PD20 derivative cell lines, PD20 (FANCD2−/−) carrying wt or K561R FANCD2, along with empty vector-transfected cells for control (Supplementary Fig. 2A). ATP5α proteins in PD20 cells carrying mtFANCD2 (inactivated) or empty vector (for control) were detected *in situ* mostly localized outside mitochondria in both stressed (H_2_O_2_ treatment) and non-stressed cells (Supplementary Fig. 2B) and both contained a similarly low amount of ATP compared to cells stably expressing wtFANCD2 (Fig. 2E). Those observations are in agreement with the finding from U2OS derivative cells (Fig. 2A–D). These results together indicate that the appropriate interaction between ATP5α and FANCD2 is essential for the function of mitochondria to generate a certain amount of ATP.

### Amino acid (AA) 42-72 of ATP5α is responsible for its interaction with FANCD2

**Figure 3:**
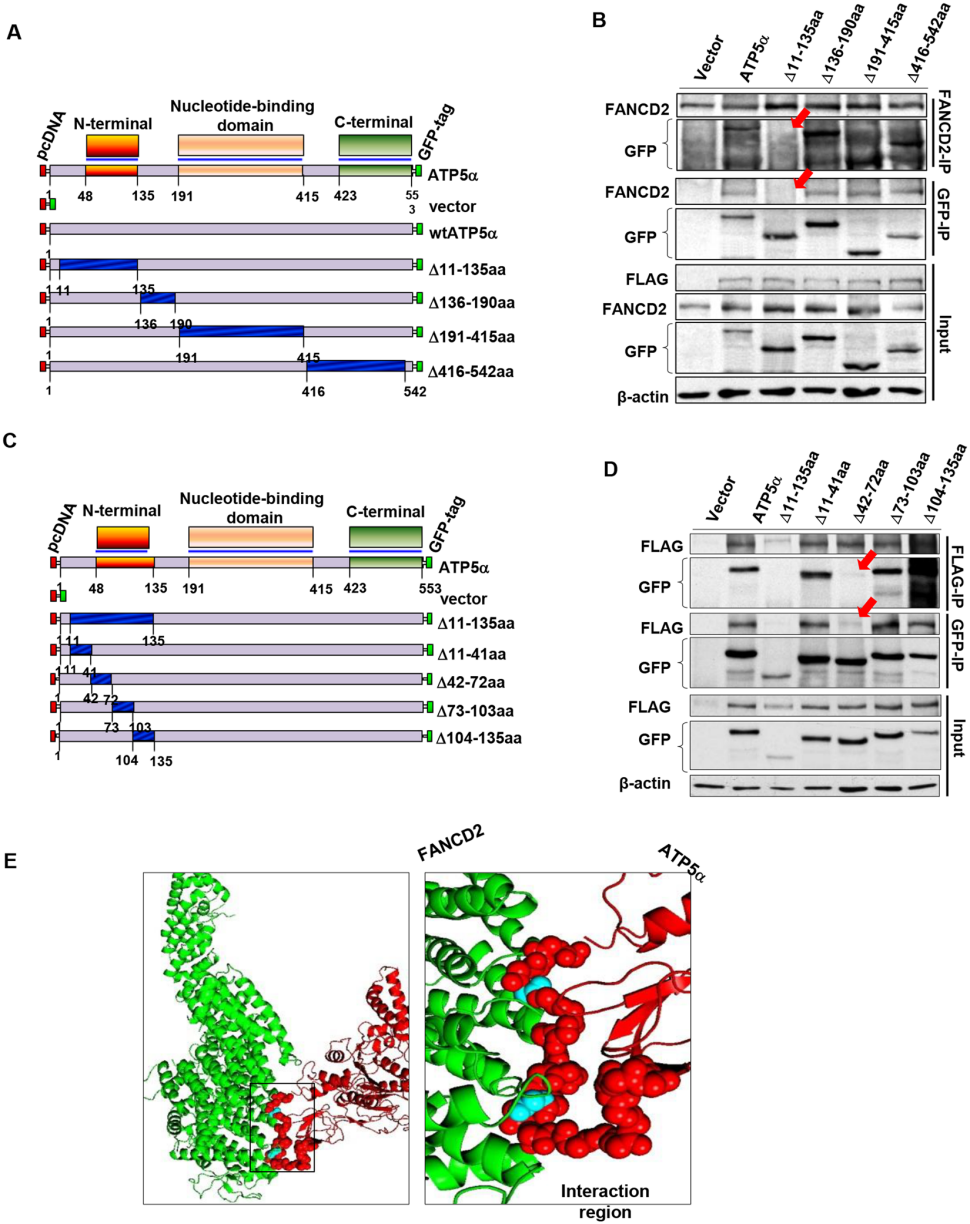
N-terminal of ATP5α is responsible for its interaction with FANCD2. (**A**) Schematic representation of a series of deletions in ATP5α protein. Each deleted part was marked with dark blue, whereas the remaining portion was shown in light gray. (**B**) Δ11-135aa of ATP5α contributes to the interaction between ATP5α and FANCD2 proteins. The six sets of reverse-IP-WB were performed using 293T cells transfected with a series of deletion constructs respectively (See Supplementary Fig. 3 for equal transfection efficiency). FANCD2 and mtGFP-ATP5α (Δ11-135aa) are not detectable in each other’s IP pellets (indicated with red arrowhead). (**C**) Schematic representation of a series of sub-deletions in the region AA11-135 of ATP5α. Each deleted part was marked with dark blue, whereas the remaining portion was shown in light gray. (**D**) AA42-72 of ATP5α is responsible for its association with FANCD2. The seven sets of reverse-IP-WB were performed using 293T cells transfected with a series of deletion constructs (See Supplementary Fig. 3 for the equal transfection efficiency), respectively. Flag or GFP was not detectable in each other’s IP pellets prepared from cells carrying GFP-mtATP5α (Δ11-135aa) or (Δ42-72aa) (indicated with red arrowheads). (**E**) The molecular docking of ATP5α and FANCD2. The predicted structure of human ATP5α (red), based on the crystalized ATP synthase subunit alpha of *bovine* (PDB: 2wss.A) using I-TASSER, adapted well to that of FANCD2 protein (green) (PDB: 3S4W.2). This specific region (42-72aa) of ATP5α interacted with FANCD2 was described as red sphere.

Next, we were interested in demonstrating the molecular details underlying the interaction between FANCD2 and ATP5α. We used an unbiased and classical domain probing approach to identify the specific region(s) that confer(s) the interaction, as illustrated in Fig. 3A. We found that mtATP5α with a deletion of AA11-135 lost its interaction with FANCD2 (Fig. 3B; Supplementary Fig. 3). To narrow down the region of ATP5α that is responsible for its interaction with FANCD2, we further dissected the region AA11-135 into 4 sub-regions (Fig. 3C) and found the sub-region AA42-72 can maintain the role played by the region AA11-135 (Fig. 3D; Supplementary Fig. 3). We further confirmed the region of ATP5α accounting for the interaction with FANCD2, by molecular docking (Fig. 3E). We utilized I-TASSER^36–38^ to mimic the protein model of ATP5α, which is structurally close to the bovine ATP synthase subunit alpha (RCSB PDB 2WSS_A) and conducted the docking with mouse FANCD2 (RCSB PDB 3S4W_B) using Z-DOCK^39^. Consistent with our data shown (Fig. 3A–D), the presence of a tight interaction between the N-terminal domain (AA 42-72) of ATP5α (Red sphere) and the second solenoid^40^ was observed to contain the monoubiquitination site (Cyan sphere) of FANCD2 (Fig. 3E). This indicated that FANCD2 monoubiquitination may be essential for ATP5α recruitment via its region AA42-72. Taken together, we not only demonstrated a previously unknown, functional aspect of FA signaling in regulating cellular ATP production but also provided in-depth molecular insights into this novel finding.

### MtATP5α (∆AA42-72) is not properly located in mitochondria, resulting in its malfunction

**Figure 4:**
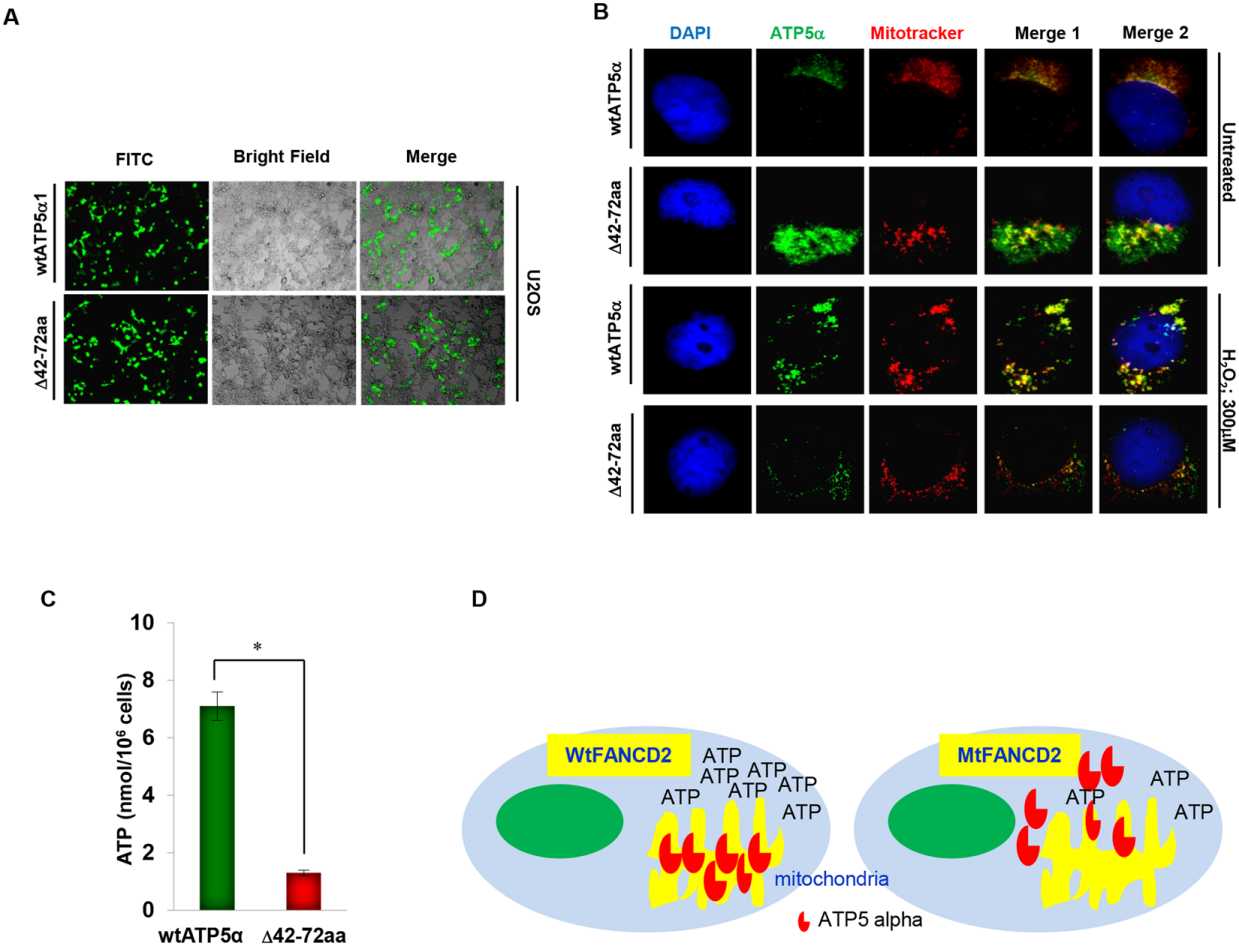
FANCD2 confers a proper ATP production via ATP5α. (**A**) Images of a stable cell pair expressing GFP-wtATP5α or mtATP5α (ΔAA42-72). (**B**) GFG-mtATP5α (ΔAA42-72) was misplaced, but not GFP-wtATP5α. The stable cells expressing GFP-wtATP5α or mtATP5α (ΔAA42-72) were incubated with MitoTracker Red CMXRos (red fluorescence) with or without H_2_O_2_ treatment. Confocal microscopy analysis was performed for visualization of red, green, or merged fluorescence. (**C**) The ATP production is low in GFP-mtATP5α-carrying cells compared to GFP-wtATP5α-containing cells. Intracellular ATP production of the stable cells expressing GFP-wtATP5α or mtATP5α (ΔAA42-72) was measured. ATP production was significantly higher (*p* < 0.005) in the wtATP5α than mtATP5α (ΔAA42-72) cells. (**D**) Schematic representation the relationship between FANCD2 and ATP5α WtFANCD2 but not the mutant (K561R FANCD2) is responsible for the location of ATP5α and the cellular ATP production.

To establish the functional importance of AA42-72 within ATP5α, responsible for the interaction with FANCD2, we decided to show biologic effects of this subject region. First, we generated U2OS derivative cells stably expressing GFP-mtATP5α with a deletion at AA42-72 and the corresponding control cells carrying GFP-wtATP5α (Fig. 4A). We then detected the localization of these two versions of GFP-ATP5α (wild type or the deletion mutant). We found that the cellular localization of GFP-mtATP5α, but not GFP-wtATP5α, showed a pattern similar to the localization of ATP5α shown in the U2OS derivative cells carrying inactivated FANCD2 (Fig. 2A) with nearly half of the detected ATP5α proteins present outside mitochondria (Fig. 4B). Similarly, the subject cells (carrying GFP-mtATP5α) also showed a reduced level of ATP production compared to their corresponding control cells (carrying GFP-wtATP5α) (Figs 2D,E and 4C). Therefore, both the N-terminal domain (AA42-72) of ATP5α and the monoubiquitination of FANCD2 at K561 play critical roles in cellular energy metabolism, enhancing our understanding of aging and cancer in general.

## Discussion

The Fanconi Anemia signaling pathway has been emerging to be a pivotal signaling network in advancing our understanding of human tumorigenesis and the effectiveness of cancer treatments^41^. The focus of this signaling network is the FANCD2 protein. In fact, FANCD2 is the only protein highly conserved across all species (from bacteria to humans), compared to many other members in the FA pathway, whose homologs are only present in mammals/vertebrates^42^. This suggests that FANCD2 may play many other roles in addition to those commonly described.

To better understand the actions of the FA signaling pathway, centering upon the monoubiquitination of FANCD2, we conducted an unbiased screening of FANCD2 functional units (FANCD2-containing protein complexes). Form this analysis, we found that ATP5α was tightly associated with wtFANCD2, but not mtFANCD2 at K561 (Fig. 1). We identified the specific region (AA42-72) within ATP5α that is essential for the interaction with wtFANCD2, and found that both mtFANCD2 (K561R) and mtATP5α (∆AA42-72)-carrying cells contained malfunctioning mitochondria and produced a significantly reduced amount of ATP (Figs 2 and 4). This observation suggested that both proteins can work in concert to achieve the appropriate mitochondrial function. One of the most prominent roles of mitochondria is to produce the energy currency of the cell, ATP, and to regulate cellular metabolism^43^. DNA damage and subsequent dysfunction in the mitochondria is an important factor in a range of human diseases due to their influence in cell metabolism. Mitochondria were initially proposed to be relevant in cancer by Otto Warburg who reported that cancer cells exhibited “aerobic-glycolysis”^2^. Although this was originally interpreted as an indication that the function of the mitochondria was defective, we now understand that cancer cells survive in an altered metabolic state^44^. To date, the functions of the FA proteins for normal cellular activities are attracting us more and more in determining cellular fates. However, it is still unclear as to if and how they utilize mitochondria as part of their tumor suppressor functions in the protection from neoplasm in human cells. This study stands as a leader to go towards the field of mitochondrial function in the context of the FA signaling.

With our recent work, we demonstrated the tumor suppressor roles of FA signaling in patients with or without FA at the metabolic level^45^. This marked us being the first to have achieved characterization of the general tumor suppressor functions of the FA signaling pathway at all three levels, genetically^46^, functionally^47^ as well as metabolically^45^. The significance of FA signaling is further supported by the occurrence of the impaired FA pathway in human cancers, in which there is about 30–50% of tumors carrying a defective signaling network at the genetic level alone. Therefore, we can imagine that the malignancy of those corresponding tumors harboring impaired FA signaling could have been accelerated by the malfunctioned mitochondria. Our study reveals new molecular mechanisms underlying the contribution of the disordered metabolism to the tumorigenicity derived from compromised FA signaling (Fig. 4D), providing fundamental understanding of human aging and cancer in general.

## Materials and Methods

### Cell lines, antibodies, and chemicals

The PD20 (FANCD2^−/−^) cell line was a gift from the FA Research Foundation. HEK293T and U2OS cells were purchased from American Type Culture Collection (ATCC, Manassas, VA) and maintained in DMEM medium supplemented with 10% fetal bovine serum at 37 °C in 5% CO2 (v/v). Antibodies against the FANCD2 and FANCL were obtained from NOVUS (Littleton, CO). The anti- ATP5α, -GFP, -vinculin and -IgG antibodies were from Santa Cruz (Dallas, TX). The anti-Flag and β-actin antibodies as well as the chemicals Hydrogen peroxide (H_2_O_2_), blue dextran, neomycin and molecular weight markers used for gel filtration were from Sigma (St. Louis, MO). MitoTracker Red CMXRos and puromycin were bought from Thermo Fisher Scientific (Waltham, MA).

### Gene knockdown by Lentivirus ShRNA

Lentiviral transduction was described previously^48, 49^. A set of five pLKO.1 plasmids containing shRNA targeting FANCL (purchased from GE Dharmacon, Lafayette, CO) along with pLKO.1 empty vector was used to generate corresponding lentiviruses. U2OS cells were then infected with these viruses according to the protocol provided. Infected cells were pool-selected with puromycin 48 hr post-infection, and FANCL knock down was verified using anti-FANCL antibodies.

### Site-directed mutagenesis and deletion constructs

As described previously^50^, full-length human ATP5α was generated by PCR amplification using pcDNA3.1/NT-GFP-TOPO TA Expression Kits (Invitrogen, Thermo Fisher Scientific, Waltham, MA) with primers (5′-ATG CTG TCC GTG CGC G-3′ and 5′-TTA AGC TTC AAA TCC AGC CAA G-3′). The deletion-mutations were generated by PCR amplification based on template pcDNA3.1/NT-GFP-TOPO ATP5α plasmid using QuikChange Site-Directed Mutagenesis Kit (Agilent Technologies, Santa Clara, CA). The sequences of primers used to generate those mutants are shown in supplementary Table 1. Plasmids were purified using Qiagen plasmid purification kits (QIAGEN Inc., Germantown, MD).

### Mass spectroscopy

The 8–16% gradient SDS-PAGE gel was used to resolve Flag-IP elutes, which were prepared from HEK293T cells transfected with Flag-containing empty vector, Flag-wtFANCD2 or -mtFANCD2 cDNA-containing plasmids. The protein gel was stained with Coomassie blue and the specific protein band were excised. The subject gel slices were further processed and subsequently analyzed by MS/MS mass spectrometry for peptide fragmentation at the Harvard Medical School Taplin Biological Mass Spectrometry Facility.

### Gel filtration

Gel filtration analysis was performed as described previously^34^. Cytoplasmic and nuclear fractions were prepared with the NE-PER Nuclear and Cytoplasmic Extraction Reagents Kit (Pierce, Thermo Fisher Scientific, Waltham, MA) following the protocols provided by the manufacturer. The nuclear extract was directly applied to a Superose 6B column equilibrated with the column running buffer containing 20 mM HEPES (pH 7.9), 200 mM NaCl, 1 mM dithiothreitol (DTT), 0.1 mM phenylmethylsulfonyl fluoride (PMSF), 5 mg/ml leupeptin, 2 mg/ml aprotinin, 0.1% NP-40 and 5% glycerol. Fractions were collected and analyzed by SDS–PAGE and immunoblotting. 2000 kDa blue dextran and 669 kDa thyroglobulin were used to determine the sizes of fractions.

### Immunofluorescence

U2OS stable cells and PD20 stable cells (1 × 10^3^) were plated and grown in sterilized black well slides for 24 h. The MitoTracker red staining was performed according to the manufacturer’s instructions. Briefly, cells were incubated under culture conditions and the MitoTracker red dye was added. After 20 min, cells were fixed with 3% paraformaldehyde and permeabilized with 0.1% Triton X-100. After blocking with 3% horse serum/PBS for 30 min at RT, and subsequently incubated with appropriate antibody at 4 °C overnight. For FANCD2 staining, anti-FANCD2 antibody was added after blocking, then incubated at 4 °C overnight as well. After washing, cells were further incubated with appropriate Alexa Fluor secondary antibody at 1:1000 dilutions for 30 min at RT. After incubation with secondary antibody, cells were washed and mounted in Vectashield mounting media with DAPI (Vector Laboratories, Burlingame, CA). Cells were observed with 10X60 lens on a Olympus BX-51microscope equipped with a SenSys fluorescence CCD camera. Images were acquired and analyzed using MetaMorph version 4.5 Premier.

### Intracellular ATP measurement

The level of Intracellular ATP was measured by using a colorimetric/fluorometric assay kit (Abcam, Cambridge, MA) according to the manufacturer’s instructions. 1 × 10^6^ cells were lysed and intracellular ATP contents were determined. The absorbance was read at 550 nm using a VICTOR Multilabel Plate Reader and ATP content was calculated based on a standard curve generated from ATP-standards on the same 96-well plate. Values were normalized to protein concentrations obtained from Bradford assays.

### Immunoprecipitation and Immunoblotting

Essentially as described previously^51^, the lysates were incubated with monoclonal anti-Flag or anti-GFP, -ATP5α antibodies overnight at 4 °C, and followed by incubation with protein A-sepharose (Invitrogen, Thermo Fisher Scientific, Waltham, MA) for 2 h. The beads were washed with IP wash buffer and then boiled in SDS-lysis buffer for 5 min. The secondary antibodies were horseradish peroxidase-conjugated anti-mouse, anti-rabbit, and anti-goat antibodies were used at a 1:10000 dilution. β-actin was used as a protein loading control.

### Statistical analysis

All values were expressed as the mean ± SD of individual samples. Samples were analyzed using Student’s t-test for two groups and ANOVA for multiple groups. A *p*-value < 0.005 was considered as statistical significance.

## Electronic supplementary material

Supplementary figure, figure legends and table
