# Supplementary material for: Involvement of FANCD2 in Energy Metabolism via ATP5α
Source: Sci Rep. 2017 Jul 7;7:4921. doi: 10.1038/s41598-017-05150-1 (PMC5501830; doi:10.1038/s41598-017-05150-1)
Supplement: Supplementary file 1 — Supplementary figure, figure legends and table [file 41598_2017_5150_MOESM1_ESM.pdf]

Supplementary Information

Involvement of FANCD2 in Energy Metabolism via ATP5α

Panneerselvam Jayabal[1,3], Chi Ma[1,3], Manoj Nepal[1,2,3], Yihang Shen[1], Raymond Che[1,2], James Turkson[1], and Peiwen Fei[1,2]*

[1]University of Hawaii Cancer Center, [2]Graduate Program of Molecular Biosciences and Bioengineering, University of Hawaii, Honolulu, HI, USA

[3]These authors equally contributed to this study.

*Correspondence and requests for the materials should be addressed to P.F. (email: pfei@hawaii.edu)

Supplementary Figure and Figure Legends

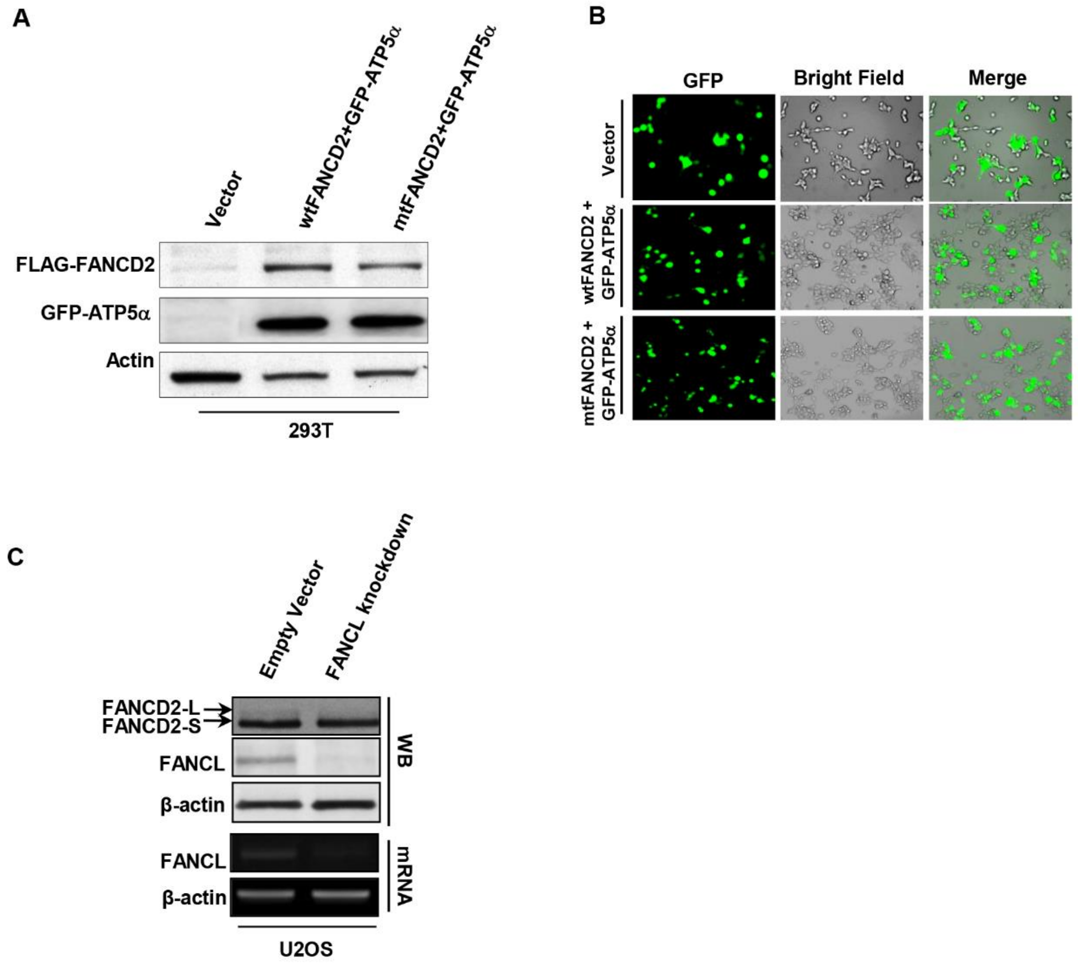

Figure S1. The transfection efficiency of 293T cells and establishment of U2OS derivative cells with or without silenced FANCL.

(A). 293T cells were transiently co-transfected with GFP-fused ATP5α and Flag-wtFANCD2 or Flag-mtFANCD2 K561R. Cell lysate were harvested and analyzed with SDS-PAGE. Then anti-Flag, GFP and β-actin antibodies were used to confirm the transfection efficiency.

(B). The images of 293T cells transiently co-transfected with GFP-fused ATP5α and Flag-wtFANCD2 or Flag-mtFANCD2 K561R showed a similar transfection efficiency among used cells to further support the Western Blot results (A).

(C). Establishment of U2OS derivative cells carrying silenced FANCL

U2OS cells were transfected with empty vector or FANCL-shRNA-carrying lenti virus, followed by selection with puromycin. The pool-selected U2OS cells were detected with anti-FANCD2, FANCL and β-actin antibodies. The mRNA samples were also prepared from the stable cells for determination of FANCL and β-actin mRNA expression.

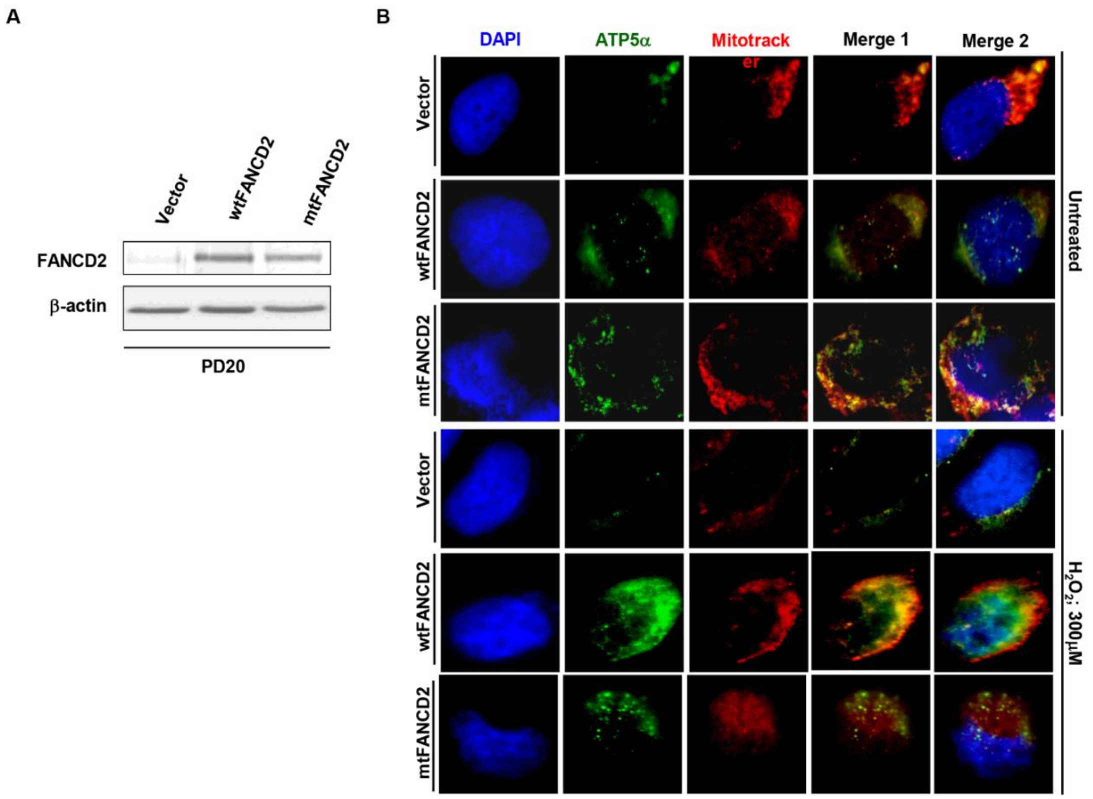

Figure S2. The establishment of PD20 derivative cells and the localization of ATP5α

(A). The established PD20 derivative cells were lysed and detected with anti-FANCD2 and β-action antibodies.

(B). Co-localization of mitochondria with ATP5α protein was in the PD20 derivative cells expressing wtFANCD2, but not in cells carrying mtFANCD2 or empty vector.

PD20 derivative cells carrying Flag-tagged wtFANCD2, mtFANCD2 or empty vector were incubated with MitoTracker Red CMXRos (red fluorescence) and goat anti-ATP5α antibodies (green fluorescence) to identify the locations of mitochondria and ATP5α respectively with or without H_2O_2 treatment. Confocal microscopy analysis was performed using appropriate filters for visualization of red, green, or combined fluorescence.

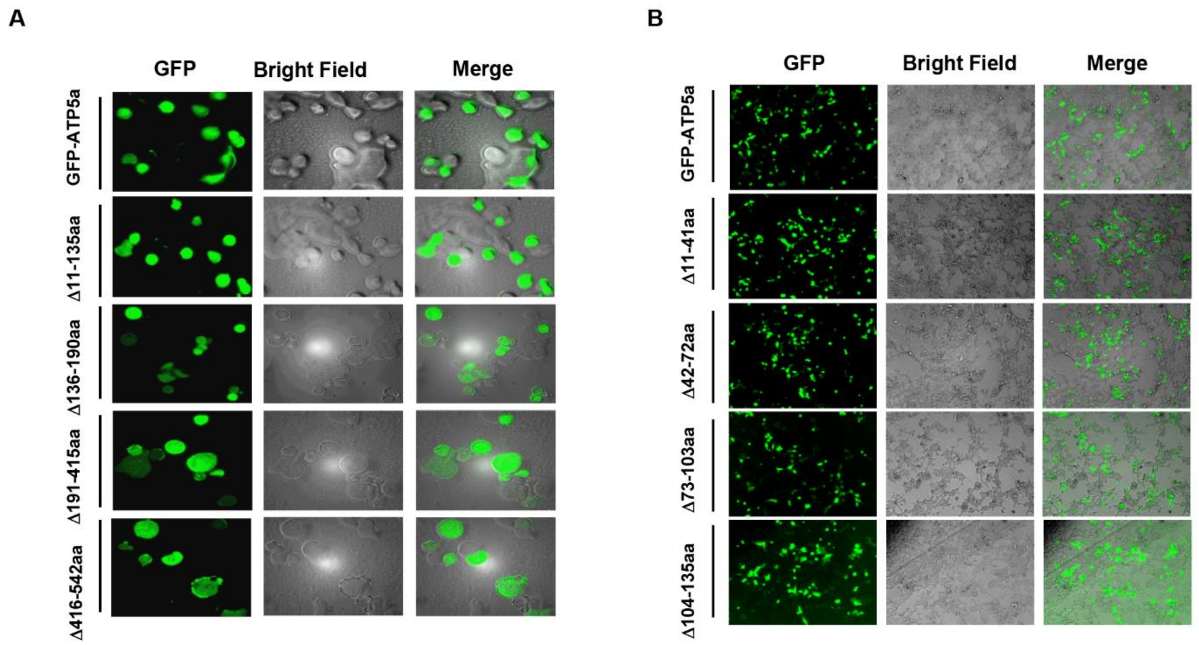

Figure S3. The transfection efficiency was equal in 293T cells transfected with wtATP5α or domain deleted ATP5α.

293T cells were transiently transfected with GFP-wtATP5α, and a series of mutants as labeled in the figure, respectively. Green cell density indicates similar transfection efficiency. Images were taken under a magnification of 400X **(A)** or 40X **(B)**.

Supplementary Table

Table S1. Mutagenesis primers for domain deletion of ATP5α

Primers	5'-DNA sequence-3'
Δ11-135aa	GTT GCT GCG GCC GTG GCC ATT GTG GAC GTT CCA<br>TGG AAC GTC CAC AAT GGC CAC GGC CGC AGC AAC
Δ136-190aa	GAT ATA GTG AAG AGG ACA GGA ACT GGC ATT AAG GCT GTG G<br>CCA CAG CCT TAA TGC CAG TTC CTG TCC TCT TCA CTA TAT C
Δ191-415aa	TGC GGG AAC CAA TGC AGC GTG TCG GAT CCG CTG<br>CAG CGG ATC CGA CAC GCT GCA TTG GTT CCC GCA
Δ416-543aa	AAC GTT GGT CTG TCT GTA TCT GTA ACA AAT TTC TTG GCT GGA<br>TCC AGC CAA GAA ATT TGT TAC AGA TAC AGA CAG ACC AAC GTT
Δ11-41aa	GTT GCT GCG GCC GTG CAT CTT CAA AAG ACT GGG ACT<br>AGT CCC AGT CTT TTG AAG ATG CAC GGC CGC AGC AAC
Δ42-72aa	CTT CCA TGC CTC TAA CAC TCG TGT CTT AAG TAT TGG TG<br>CAC CAA TAC TTA AGA CAC GAG TGT TAG AGG CAT GGA AG
Δ73-103aa	TGA TCT TGA AGA AAC TGG GGG TAT GTC CTT GAA CTT G<br>CAA GTT CAA GGA CAT ACC CCC AGT TTC TTC AAG ATC A
Δ104-135aa	TTT TCT TCA GGC TTA AAG GCC ATT GTG GAC GTT C<br>GAA CGT CCA CAA TGG CCT TTA AGC CTG AAG AAA A
